# The Hospital and the Surgeon

**Published:** 1916-11-11

**Authors:** S. S. Goldwater

**Affiliations:** Superintendent Mount Sinai Hospital, New York.


### THE HOSPITAL AND THE SURGEON.*

### II. SERVICE AND ADMINISTRATION.

By S. S. GOLDWATER, M.D., Superintendent Mount Sinai Hospital, New York.

(*Continued from* p. 88.)

In the hospital, as well as in the dispensary, the surgeon usually works without salary or fee. While the hospital surgeon sacrifices time and strength to the needs of ward patients, his sacrifices are offset by the opportunities that his position brings to him. To succeed in surgery without a hospital appointment is an uphill fight; as surgical work increases in complexity and demands increasingly the support of other diagnostic and therapeutic departments, it becomes more and more difficult for a surgeon to practise independently of a hospital. In the hospital the surgeon has consultants at command; he likewise commands the facilities of the laboratory and of the $x$-ray department, and the invaluable services of the resident medical and nursing staffs. The prevalent keen competition for unsalaried hospital positions indicates clearly enough that the acceptance of such a position is not an act of unadulterated self-sacrifice, and that indirect rewards are hoped for.

### A Question of Principle.

The hospital may experience difficulty in enforcing discipline on a surgeon who must get his living outside of the hospital. This situation must be faced frankly. Conceding that the strict enforcement of hospital regulations does occasionally work great hardship on the visiting surgeon, the primary consideration is to provide adequate care for the hospital's patients. If this cannot be obtained from the assigned visiting staff, a new unpaid staff must be substituted which can and will give the required service, or the system must be changed. Changes in system have already been tentatively made in a number of notable instances. The change which is most commonly urged is the substitution of a full staff of salaried visiting surgeons for an unsalaried staff; a modification of this system is the appointment of a full-time salaried chief of service to direct the work of the unpaid subordinate attending surgeons.

The appointment of full-time salaried visiting surgeons has been effected in certain cases not so much for the purpose of ensuring adequate attention to hospital patients as to promote research and to develop teaching. Hospitals which have thus committed themselves to a policy of research and education have been interested not only in the highest development of their own staffs, but in the education of undergraduate students, an attitude which is worthy of commendation. Much emphasis is placed nowadays on the clinical training of the medical student, and it is obvious that such training cannot be given by a medical school which does not command the facilities of a suitable hospital. Hospitals that disinterestedly lend themselves to the purposes of medical education are pursuing a wise social policy, and are serving many patients besides those in their wards.

Salaries may be paid to visiting surgeons for less than full-time service. The trustees of the most important municipal hospital in New York City are committed to the payment of salaries to the chiefs of their medical and surgical services, respectively, for a minimum of four hours of daily service. This policy has been advocated on the ground that the required service cannot be obtained on any other terms, a claim which it is difficult to understand in view of the fact that other New York hospitals possessing no greater attraction to surgeons of high standing have no difficulty in obtaining the men needed or in holding them to the observance of exacting rules. To assert, however, that it is possible for such hospitals to obtain service gratuitously is not to argue against the justice of paying salaries to the visiting staff; the ethics of the proposal is one thing—its necessity or expediency is quite another.

### The Private Pay Ward.

Reference has been made to two forms of compensation—namely, financial compensation and compensation in opportunity. There is a third form of compensation which has, perhaps, an indirect money value. I refer to the facilities which are placed at the disposal of the surgeon by hospitals which care for both free and private patients. If the surgeon's principal income is derived from the operations which he performs on well-to-do patients, it is of value to him to be able to offer to such patients the facilities of a well-equipped private hospital. Furthermore, if he is to give his services gratuitously to ward patients, it is advantageous to be able to do so with a minimum expenditure of time and effort. Both of these objects are realised when the hospital places at the disposal of the surgeon a well-equipped private ward in close proximity to, if not under the same roof with, the public wards in which he spends part of each working day.

The choice of the assistant visiting staff rests usually with the hospital management rather than with the visiting surgeon. On the supposition that hospital control is best entrusted to an impartial lay board, one must assent to the choice of the visiting surgeon's assistants by the trustees. I have already pointed out the possibility of a system of co-operation with the medical staff in the nomination of eligible candidates for the position of visiting surgeon which, while reserving to the trustees the

* We are indebted to the courtesy of Dr. Goldwater and the *Modern Hospital* for the copy of this paper, which was read before the American Hospital Conference, Philadelphia, September 1916.

final choice, nevertheless ensures due regard for enlightened professional opinion. The system described may advantageously be followed, also, in the appointment of assistant visting surgeons, with the proviso that the medical men serving on the conference committee shall include the chief of the service with which the assistant visiting surgeon is to be identified.

The hospital surgeon may be aided or hindered by the pathological department. For the sake of both surgeon and patient, the hospital is bound to provide a well-equipped and well-manned pathological department. In hospitals having a large surgical service it is desirable that the equipment necessary for the preparation of sections for diagnostic purposes be provided in a room adjoining the operating room, and that a pathologist be at hand during operating hours. Apart from the performance of routine diagnostic work, the laboratory must be ready to prepare microscopic sections extensively for study, must be ready to perform autopsies promptly and satisfactorily, and should make it a point to arrange such autopsies at a time which is convenient to the surgeons concerned, since a *post-mortem* examination in a surgical case loses much of its value to the hospital if performed in the absence of the surgeon.

### The X-Ray Department.

From the standpoint of diagnosis the importance of the radiographic department to the surgeon is inestimable. Hospital radiology has been seriously handicapped because of the hesitancy of hospitals to devote large sums to the support of this comparatively new department. There is no logical reason why the radiographic department should be singled out as one to be placed on a self-supporting basis—a poor patient is no less deserving because he happens to require a costly *x*-ray examination. It is significant, however, that in almost every instance in which a hard-pressed hospital has endeavoured to make its *x*-ray department self-supporting, the effort has succeeded. From the surgical standpoint it is essential that the radiographic department be properly equipped and organised, and experience has shown that, by one means or another, it is practicable to accomplish this in any hospital.

No surgical service can be regarded as satisfactorily conducted which does not systematically consult with an internist as well as with other specialists, medical or surgical, whose advice may be required. Experience has shown that a consulting staff which does not visit the hospital daily is not as serviceable to the surgeon as a regular visiting staff. The surgeon who is most advantageously placed (it would perhaps be more logical to speak of the advantageous position of the patient) is one who is connected with a general hospital the organisation of which includes representatives of all the recognised branches of medicine and surgery.

It is a matter of common observation that clinical records are not as complete in surgical as in medical wards. Whether clinical records are well or ill kept depends primarily on the surgeon. But while the making of the record depends on the surgeon and on his control over his assistants, the filing of the record is a matter of hospital administration. In this department centralisation is the avenue to success. In large cities a patient may be successively treated in a number of institutions. When this occurs, the hospital which was first to handle the case owes a complete record to the institution which undertakes the later treatment. Reciprocity in these matters is now satisfactorily established in many communities, but satisfactory service is not feasible in the absence of a suitable organisation in the record-room. That there can be no satisfactory collation of clinical data for scientific study without proper filing and indexing is obvious. In the choice of nomenclature, standard classifications which are in general use should be preferred.

The interests of surgery itself demand that the surgical service of the hospital shall be of the "continuous" variety. This does not mean that the chief of staff must be on actual duty every day in the year, but that his control over the service shall be continuous; due allowances must, of course, be made for necessary periods of recreation, including periods for rest, travel, and study. The most successful surgical organisation in the United States is perhaps the least self-centred. Its members make a point of informing themselves concerning the activities of other clinics throughout the country and abroad; this is accomplished by means of travelling representatives. This spirit of interest and method of observation are worthy of emulation. A disposition on the part of the surgical staff to keep in touch with the work of other surgical clinics should be freely encouraged by the hospital. Scientific progress can be promoted in large hospitals by permitting and encouraging individual surgeons to concentrate on certain types of cases. Such a segregation of surgical material may involve a departure from the fixed form of the hospital's organisation, and this may result in personal opposition to the plan; but such opposition cannot be defended if all the members of the staff are granted equal opportunities.

The hospital should place at the disposal of the surgical staff operating rooms which are sufficient in number and equipment, with an adequate personal organisation. The number of operating rooms cannot be fixed by rule, but must be determined by the requirements in each case. The size of the operating plant of a hospital maintaining a given number of surgical beds should be calculated with relation to the proportion of ward and private beds, the number of emergency cases treated, the number and organisation of the resident staff and of other available assistants. For a given number of ward cases, in the hands of a small staff, fewer operating rooms are required than for the same number of private patients treated by many different surgeons. The facilities should be such as to permit of the prompt treatment of all emergency cases. Thus, if it be estimated that an average of five or six surgical cases will be treated daily, and that these can be handled successively in a single operating room, a second room should nevertheless be available for emergency use.

What has just been said indicates that, in the very planning of the hospital buildings, a surgical service may be made or marred. The plan of the surgical ward very materially affects not alone the convenience of the surgeon, but the comfort of the patient. A large ward designed for an acute surgical service should have recovery rooms, where post-operative cases may be cared for without causing distress to other patients; separation rooms for moribund or delirious patients, or for patients with foul wounds; rooms where convalescents may take their meals amid appropriate surroundings; rooms or wards for surgically complicated contagious cases; and balconies or roof gardens to which convalescents may resort when out of bed and where septic cases may receive fresh-air treatment.

### Surgical Statistics and Results.

The investigation of surgical end-results is a matter of serious importance. The employment of special workers to carry on end-result investigations in the field is now the practice of a number of hospitals. Despite the cost involved, such proposals should be sympathetically received by hospital administrators. The hospital is one of the greatest of existing social institutions, supported in this country at a total cost which staggers the imagination. *The value of its output must not be assumed, but must be proved.* The altruistic hospital surgeon will not permit his equanimity to be disturbed by the revelations of end-result investigations; if the disclosures are sometimes surprising, they are always instructive.

I have indicated many ways in which the hospital can and should assist the surgeon. Let me now turn to another aspect of the relations between the hospital and the surgeon. Superintendents assert, and surgeons do not deny, that the activities of surgeons need to be regulated; phases of regulation will therefore be considered.

The hospital owes certain duties to the sick poor. One of these duties is to conduct its admissions in a strictly impartial manner. Medical urgency and not personal influence should determine the order of the admission of patients.

While the hospital is endeavouring to fulfil its duty toward the public, the surgeon is endeavouring —in a similar fashion, but on a different plane— to take care of his own *clientèle*. The surgeon's *clientèle* is more limited than that of the hospital, and the surgeon can hardly be blamed if he does not take cognisance of needs which are beyond his ken. The surgeon very naturally looks to the hospital to care promptly for patients in whom he is personally interested, and in his eagerness to serve these patients he brings to bear on the admitting officer of the hospital the heavy pressure which his office and personality enable him to exert. To such pressure the hospital cannot conscientiously yield. The argument is sometimes made that the hospital should give preference to patients in whom its visiting surgeon is interested, by way of compensating the surgeon for his hospital work. This argument is fallacious. If the hospital owes anything to the surgeon it has no right to discharge its debt at the expense of the friendless poor.

In the matter of the discharge of patients, as well as in connection with their admission, it is at times necessary for the hospital to take cognisance of and to regulate the activities of its staff. The surgeon who is zealous in the pursuit of science will naturally take greater interest in some patients than in others. A patient who, from the scientific point of view, is regarded as undesirable, may nevertheless have a strong claim on the care of the hospital. Influenced, perhaps unconsciously, by the trend of his scientific interest, the surgeon may propose to rid his service of a patient who should not be discharged.

When the hospital is working under such pressure that beds cannot be provided promptly for applicants in whom the visiting staff are interested, the attending surgeon, quite without culpable intent, may persuade himself that the period of hospital treatment has been unduly prolonged—that laparotomy cases, for example, can be safely ordered out of bed within an incredibly short time after operation. Whatever the merit of the surgeon's point of view, the hospital administrator is bound to consider independently the justice and the wisdom of discharging each patient. The hospital may and should take cognisance of social facts which the surgeon cannot be expected to know or to consider. The foregoing remarks must not be interpreted as signifying a belief on the part of the writer that in these matters surgeons are guilty of moral or mental obliquity. They are, as a rule, normal human beings, whose minds functionate like the minds of other human beings. The surgeon simply has the defects of his very valuable qualities; his work is intensive, and his field of vision is apt to be narrowed accordingly.

In most hospitals routine operative work is begun at a specified hour, which may be a morning or an afternoon hour. If it is the prevailing practice of the surgeons of the community to attend to office practice in the morning the hospital surgeon will prefer to attend to his hospital operative work in the afternoon; yet the very surgeon who adopts this *régime* may express a preference for early morning as time of choice for grave surgical operations in the case of private patients. There is an inconsistency here which demands attention. If the surgeon believes that there are physiological or clinical grounds for a certain choice of hour, by what right does he deliberately arrange to operate on hundreds of ward cases at a less advantageous time? From the standpoint of hospital administration, the proper way to deal with this question is to ascertain the consensus of authoritative opinion, and to act accordingly.

Conflict between the hospital's interests and the private interests of the surgeon occasionally takes the form of the postponement of an operation on a ward patient after preparation—in other words, after a day of starvation and anxiety. There may be sound reasons for the postponement of an operation for which preparations have been made; but, when the reason given is no more serious than a social engagement, the hospital is bound to protest. It must not be inferred that incidents of this kind are frequent, or that in the belief of the writer surgeons generally put their own comfort and convenience before the interests of their patients; the contrary is the rule.

In the management of the hospital's surgical service—a minor matter, but one which demands attention—is the right of interns, operating-room nurses, and other employees to regular meal-hours. The physical and mental strain associated with surgical operations is not confined to the chief surgeon. His assistants, each striving to perform the duty assigned to him, are frequently subjected to very severe strains. Emergency work is for ever turning up in operating rooms at uncomfortable hours, and assistants are compelled to adapt themselves to the situation. As a rule, they do so ungrudgingly; but their sacrifices must not be lightly passed by. Such sacrifices may be sanctioned when their purpose is to save life or to alleviate suffering, but not in order to enable the visiting surgeon to keep a dinner engagement.

### The Age Limit.

In the larger cities hospital after hospital is recognising the principle of the age limit for the visiting staff. Some ten years ago a hospital which was about to be reorganised sought, by means of a widely distributed *questionnaire*, to ascertain the extent to which hospitals in this country had undertaken to retire members of their visiting staffs on account of age. Although an age-limit rule had long been in force in the public hospitals of France, in the leading voluntary hospitals of Great Britain, and in the Medical Service of the United States army and navy, it was ascertained that neither the municipal nor the voluntary hospitals of this country had yet given serious consideration to this question. Since then much progress has been made. There are few important hospitals in the country to-day which have not either actually adopted an age-limit rule or have not seriously considered doing so.

I have already expressed my preference for the staff hospital in large cities. I am not blind, however, to the interests of the general profession, nor lacking in sympathy for the physician or surgeon who has no hospital connection. While in large cities it may be necessary and in the interest of surgical efficiency to organise hospital staffs along lines which seem to the excluded outsider to be monopolistic and unfair, it does not follow that consideration should not be shown to men outside of the ranks.

A staff hospital which does not maintain the highest surgical standards cannot justify itself; nor can a staff hospital be held blameless if it does not train interns in excess of the number required to keep the staff full; each year men should be sent out prepared to take up hospital duty in the new institutions which are springing up everywhere, or ready, if need be, to perform safe and sane surgery apart from hospitals, especially in rural communities which lack hospital facilities. The staff hospital should adapt itself to the requirements of the medical school, if there be one in the community, so that, besides training interns to become surgeons, it may afford to undergraduate students, whose fortune it may never be to become surgical interns, some slight insight into surgical technic, some appreciation of the gravity of surgical operations, and a wholesome distaste for undertaking important surgical work without adequate training.

### The Hospital and the General Practitioner.

That a staff hospital, well organised and well equipped, interferes with the earnings of the general practitioner, cannot be denied. The assertion is frequently made that the hospital competes, even that it competes unfairly, with the general practitioner. If those who advance this argument mean that a well-organised and well-equipped hospital offers to the patient, and especially to the poor patient, an excellence of treatment and a measure of safety which he cannot have in his own home or so long as he remains in the care of his family physician, the charge is true. It is far from true, however, that hospitals and hospital surgeons compete with the general practitioner in the sense that there exists between the two groups a conflict of interest and an open hostility. The charge that such hostility exists is not in accordance with the facts. In my own experience the surgical wards of a large hospital have been occupied almost exclusively by patients referred to the hospital, or to members of the staff, by general practitioners. Any competition that exists, therefore, does not exist between the hospital and the general practitioner, or between the surgical staff and the general practitioner, but between the physician's honest appreciation of the needs of his patient and his—*i.e.*, the physician's—economic interests. It is to the credit of physicians that in this competition their higher instincts are almost invariably victorious, and that hospital wards are filled with surgical cases through the honesty and self-denial of physicians who, rather than undertake work which they are not qualified to perform, deny themselves the privilege and profit of performing surgical operations at home, and place at the disposal of their patients all that a well-equipped hospital has to offer.

What can the hospital do for the general practitioner in return for his co-operation and support? It can make him welcome in its wards; it can co-operate with him by diagnosing, without charge or for a moderate fee, complicated cases requiring the combined study of various members of the staff; it can keep him informed concerning the admission and discharge of his patients; it can, where required, offer the use of its laboratories, its *x*-ray department, its departments for special therapy; it can conduct post-graduate courses for his benefit; and last, but not least, it can perform for him and for his patients a simple act of justice by selecting its surgical staff with an eye single to efficiency and public service.

